# Patient Engagement in a Virtual Cardiac Rehabilitation Platform: Secondary Analysis of the ERA-Nudge Trial

**DOI:** 10.2196/64502

**Published:** 2026-11-06

**Authors:** Prerna Gupta, Josh Durfee, Joy Waughtal, Dana R Fletcher, Vincenzo B Polsinelli, Harsh Vathsangam, Joan F Hilton, Alexis L Beatty, P Michael Ho, Larry A Allen, Sheana S Bull, Pamela N Peterson

**Affiliations:** 1 Division of Cardiology, Department of Medicine, University of California, San Francisco, 555 Mission Bay Boulevard S, San Francisco, CA, 94158, United States; 2 Center for Health Systems Research, Denver Health, Denver, CO, United States; 3 mHealth Impact Lab, Department of Community and Behavioral Health, Colorado School of Public Health, Aurora, CO, United States; 4 Evida Research, Denver, CO, United States; 5 Division of Cardiology, Department of Medicine, University of Colorado Anschutz, Aurora, CO, United States; 6 Movn Health, Irvine, CA, United States

**Keywords:** cardiac rehabilitation, mobile health, mHealth, patient engagement, mobile health intervention, healthy behaviors, application, randomized trial, mobile app

## Abstract

**Background:**

Mobile health interventions have the potential to motivate healthy behaviors. Optimal strategies for engaging patients in cardiac rehabilitation (CR) through mobile health interventions remain unclear.

**Objective:**

This study aimed to examine patient engagement in a mobile app platform for CR across patient characteristics and by the method of delivery of nudge messages over the 12 weeks of CR.

**Methods:**

The authors analyzed data from the ERA-Nudge (Enhancing Rehabilitation Adherence Through Optimized Nudge) study, a pragmatic randomized trial conducted at an urban safety-net hospital testing nudges (short messages designed using behavioral economics to elicit behavior change) to enhance CR adherence and log healthy activity. Trial patients were given access to a smartphone-based app. Nudges were initially sent as app notifications. Later in the study, patients received nudges as text messages. We assessed patient characteristics associated with app engagement, and we compared time spent in the CR app among patients receiving nudges as app notifications vs text messages. The trial enrolled patients who could engage with messages in either English or Spanish at an urban safety-net hospital with a qualifying CR diagnosis and a smartphone from May 2019 to May 2023. We describe minutes spent in the app across patient characteristics and by type of nudge delivery, and we compare the 2 approaches using unadjusted and multivariate negative binomial regression over the 12 weeks of CR.

**Results:**

In total, 223 patients received nudges: 74 via app notification and 149 via text message. Overall, patients spent an average of 219 (SD 328) minutes in the app, and 3 (1.3%) patients used the app for zero minutes. Patients receiving only app nudges spent a mean of 167 (SD 365) minutes in the app, and patients receiving text messages and app nudges spent 245 (SD 306) minutes in the app (unadjusted ratio 1.5, 95% CI 1.1-2.0; *P*=.02). After adjusting for age category, sex, primary language, CR location (hospital vs home), and highest education achieved, Spanish-preferring patients, patients with some college education, and patients receiving text messages spent more time in the app. Patients receiving nudges as text messages and app notifications spent an adjusted ratio of 1.8 (95% CI 1.2-2.5; *P*=.002) times as many minutes in the app compared to those getting nudges as app notifications.

**Conclusions:**

Among an urban safety-net population, text message nudges were associated with greater time in the app compared to nudges delivered as app notifications. These findings suggest that text messages may result in greater patient engagement for mobile health interventions.

## Introduction

Disparities in access to and availability of cardiovascular care persist. Burgeoning mobile health (mHealth) technologies have the potential to augment patient care by improving care team interaction and motivating healthy behavior change. An important use case is cardiac rehabilitation (CR). CR portends immense benefits for patients [1-6], but referral rates are approximately 30%, and participation rates are even lower at 8% to 9% [1,2,5,7]. Numerous barriers to CR attendance lower adherence rates, and augmentation of CR delivery via mobile tools such as telehealth CR or a mobile app may circumvent these obstacles [2,5]. There are numerous platforms in development, but clear benefits from these mobile or hybrid CR interventions have been inconsistent [8]. Approximately half of virtual CR alternatives report improved adherence, physical activity, or functional status. Reasons for the mixed results are underexplored, and patient use of the technologies is infrequently reported. Heterogeneity in patient use across studies may be a reason for inconsistent outcomes, but strategies to best engage patients remain unclear [2,9].

A possible explanation for inconsistent improvement in outcomes of virtual CR trials could be the lack of use by patients—like medications, patients must use interventions for them to work [8]. Some studies of hybrid or remote CR have resulted in fewer adherences compared to traditional CR, and the mechanisms of this suboptimal adherence are undefined. One way to engage patients may be to send nudge messages designed to create behavior change using principles of behavioral economics [10,11]. It is not yet known how these nudge messages should be delivered or how they may impact patient engagement.

To better understand how to engage patients, the primary objective of this analysis was to identify patient characteristics associated with patient engagement and compare patient engagement in the ERA-Nudge (Enhancing Rehabilitation Adherence Through Optimized Nudge) study of an app-based CR platform based on whether patients received nudges via both text messages and app notifications or app notifications alone, as assessed by the amount of time spent in the app. While most CR studies evaluate attendance at CR or exercise sessions by CR patients, we aimed to assess not the exercise minutes or sessions, but rather patients’ engagement with mobile technology as measured by time spent in the app when patients have a virtual CR option. The ERA-Nudge study aims to improve referral and adherence to CR using short messages designed to elicit behavior change or nudges [10,11]. Nudges were initially sent as notifications within a mobile phone app, Movn (Movn Health), and later also sent as text messages. We identified patient characteristics associated with app engagement and evaluated the intervention factor of text messaging to determine if it was associated with app engagement independent of patient factors, with the hypothesis that text messages may be associated with greater engagement with the app.

## Methods

### Population

This is a secondary analysis of the ERA Nudge study (NCT03834155). The protocol has been previously published [12]. Briefly, this study enrolled patients with a qualifying CR diagnosis who speak English or Spanish and have access to a smartphone. Patients were identified daily from the hospital census based on a qualifying diagnosis by the CR team. The trial was a single-center study conducted at Denver Health, an urban safety-net hospital with over 60% of patients identifying as minorities, and it enrolled patients from May 2019 to May 2023. The app usage data were sourced from Movn Health, the company that delivers the app, and the remainder of the data was compiled from the electronic health record and collected trial data. For the purposes of our study, we evaluated the time spent in the app by patients receiving nudges as either app notifications or text messages.

### Ethical Considerations

All eligible patients provided consent prior to enrollment and randomization. The trial protocol and analysis plan were approved by the Denver Health and Colorado Multiple Institutional Review Board (18‐1290). Data were deidentified prior to analysis. Compensation was provided to participants for completing surveys. Participants received a US $25 gift card for completing each study assessment.

### Movn App

As part of the trial, all enrolled patients were given access to the Movn app on their smartphones. This platform facilitated chat-based communication between the CR team and patients, provided educational resources, and enabled patients to log exercise type and duration. The app also allowed for patient data entry of weight and vital signs such as heart rate and blood pressure. The app was intended for data logging, chat, and the provision of educational resources. The patients’ time spent in the app may not have included the time they spent exercising, as these data were logged after an exercise session. These data were not required, and patients were not provided with exercise equipment, but they were able to enter this information if they had equipment. Movn Health collected data on how many minutes each patient spent in the app from the program start date to the graduation date. All patients were trained in app use upon enrollment in the trial by study staff.

### Trial Design

The ERA-Nudge study consisted of a 2×2 factorial design, randomizing patients to (1) choice of hospital or home-based CR vs hospital-based CR and (2) optimized nudges vs no reminders. We developed nudges through iterative focus group sessions and designed them to encourage healthy behavior aligned with general messages around CR. The messages encouraged healthy behaviors but did not specifically include information on how to use the app. They were sent once or twice per week, and only to patients randomized to receive nudges. Nudges were initially sent as app notifications and into the patient’s inbox within the app. However, by July 2021, patients expressed to study staff that they were not seeing the nudges. In response to this feedback, study staff added text messages via a mobile messenger platform, providing the same nudges on the same schedule in both messaging formats, thereby enabling patients to view the messages as an app notification or a text message. Given that nudges were in the patients’ app inboxes, the nudges were always available for viewing within the app, regardless of participants’ app notification settings. Patients’ settings regarding app notifications were not viewable or available to Movn or study staff. The nudge messages and schedule remained the same. We aim to evaluate whether the nudge delivery modality was associated with greater use or time spent in the app among patients receiving nudges—whether patients interacted with the app more when they received text message or app notification nudges. Apart from the switch from app notification nudges to text message notifications, all other methods were held constant, including app training protocols and trial enrollment. Based on feedback during the initial app notification nudges phase, no changes were made in the app training protocol for patients who received text message nudges. Given our study focuses on nudge message modality, our cohort comprises exclusively patients receiving nudges.

All patients referred to CR were screened for the trial. Eligible patients were then consented and enrolled by study staff. As part of the initial intake, patients also provided their educational status, employment, income, and Patient Health Questionnaire-9 (PHQ-9), an instrument used to assess depression. Clinical data, such as qualifying conditions, were collected by study staff based on the electronic health record.

### Statistical Analysis

We evaluated baseline patient factors using the mean and SD for continuous variables and proportions for categorical variables. Demographics, clinical indication for CR, and CR location were compared among subgroups using chi-square tests or Fisher exact tests, where appropriate. We performed unadjusted comparisons of each covariate with the outcome variable, minutes in the app, as well as an adjusted analysis accounting for all patient factors. We used a negative binomial regression with a log-link function to account for the zero-weighted, skewed data in the full cohort. The final multivariable model was determined based on a prespecified approach incorporating age category, sex, CR location (home vs hospital), and the primary exposure variable: text message and app notification nudges vs app notification nudges alone. Educational status, employment, and income demonstrated significant collinearity. For the determination of the final multivariable model, multivariable models incorporating educational status, employment, or income were compared using information criteria to arrive at the final multivariable model. Interaction terms were evaluated, but none were significant. The resulting ratios identify the ratio of minutes in the app between the compared groups. Results are reported according to the STROBE (Strengthening the Reporting of Observational Studies in Epidemiology) checklist (Checklist 1). All analyses were performed using SAS Studio (version 3.8.1; SAS Institute).

## Results

### Baseline Characteristics

Overall, in the ERA-Nudge study, 447 patients were enrolled and randomized (224 to nudges and 223 to no nudges). Among these, 1 patient withdrew during the baseline visit, resulting in a cohort of 223 patients for this analysis (Figure 1). Among our cohort of patients for this analysis receiving nudges, 74 patients received nudges as app notifications only, and 149 patients received both text message and app notification nudges.

Our cohort includes 167 (75%) male and 56 (25%) female patients. The average age of the cohort was 55.5 (SD 11.5) years. Additionally, the trial cohort included 153 (69%) White patients, 36 (16%) Black patients, and 7 (3%) patients of other races. The cohort included 88 (40%) Hispanic patients and 55 (25%) patients who preferred Spanish over English. There were 75 (34%) patients who participated in CR at home. Of note, 95 (43%) patients had an income level of US $20,000 or less, and 99 (44%) patients in the cohort were employed at least part-time (Table 1). The most common qualifying condition in our cohort was percutaneous coronary intervention (n=96, 43% in the whole cohort). Other qualifying conditions included patients with acute coronary syndrome (n=87, 39%) and heart failure (HF; n=56, 25%). Data on intake demonstrated that 87 patients (52% of 167 total PHQ-9 tests gathered at baseline) had PHQ-9 scores greater than 4, consistent with at least mild depression.

Patients receiving text messages had a mean age of 54.7 (SD 12.4) years; 73% were male (109/149), 62% were White patients (93/149), and 36% received CR at home (53/149). Patients receiving app notifications had a mean age of 57.3 (SD 9.4) years, 78% (58/74) were male, 81% (60/74) were White patients, and 30% (53/74) received CR at home (Table 1).

Overall, the distribution of minutes spent in the app among the cohort was skewed toward nonuse (Figures 2 and 3). Overall, patients spent an average of 219 (SD 328) minutes in the app. There were 3 out of 223 (1.3%) patients who used the app for zero minutes.

**Figure 1.**
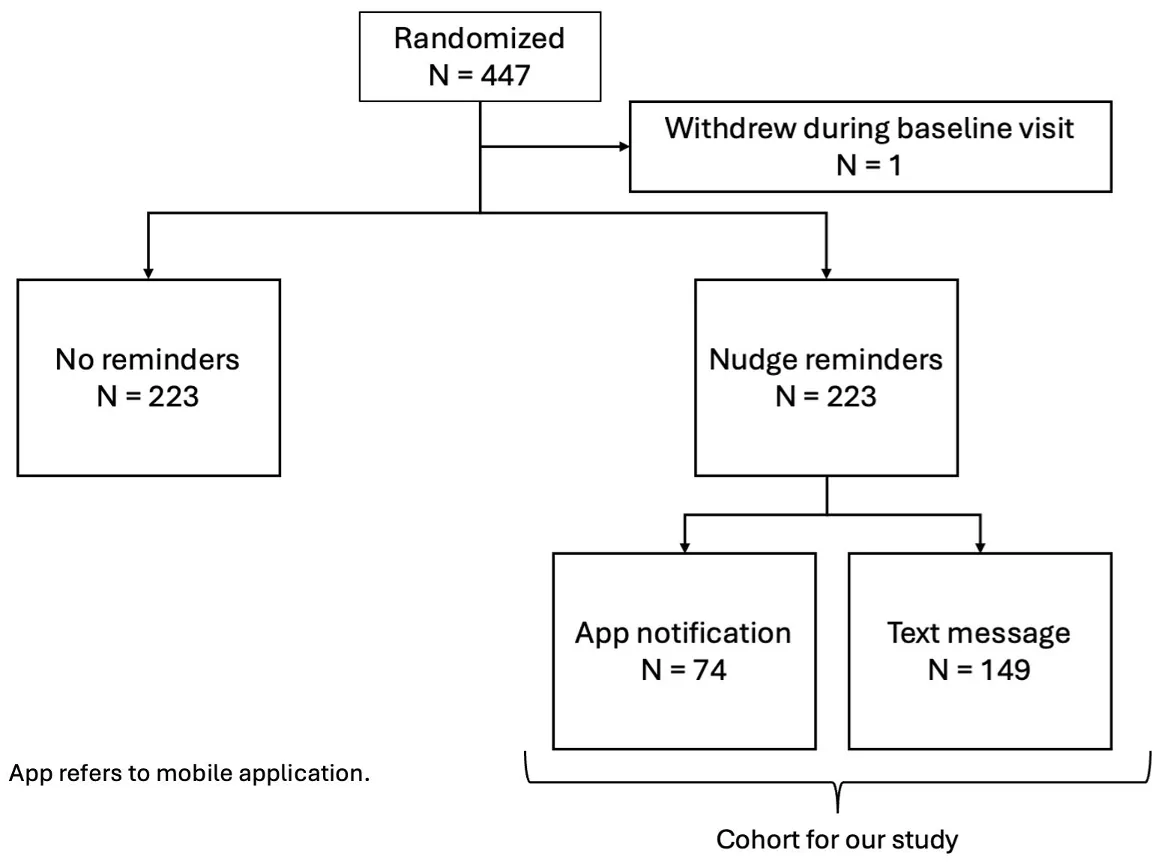
CONSORT (Consolidated Standards of Reporting Trials) flow diagram.

**Table 1.** Demographics, socioeconomic status, and clinical indications among study participants in the text message and app notification groups^a^.

Characteristic	Overall (N=223)	Text message (n=149)	App notification (n=74)	*P* value
Age (y), mean (SD)	55.5 (11.5)	54.7 (12.4)	57.3 (9.4)	.09
Age tertile (y), n (%)				.13
20‐51	72 (32)	54 (36)	18 (24)	
52‐61	79 (35)	47 (32)	32 (43)	
62‐85	72 (32)	48 (32)	24 (32)	
Sex, n (%)				.49
Male	167 (75)	109 (73)	58 (78)	
Female	56 (25)	40 (27)	16 (22)	
Race, n (%)				.03
White	153 (69)	93 (62)	60 (81)	
Black	36 (16)	28 (19)	8 (11)	
Other	7 (3)	5 (3)	2 (3)	
Unknown or not reported	27 (12)	23 (15)	4 (5)	
Hispanic, n (%)	88 (40)	59 (40)	29 (39)	.88
Spanish, n (%)	55 (25)	36 (24)	19 (26)	.87
Home-based CR^b^, n (%)	75 (34)	53 (36)	22 (30)	.38
Level of education, n (%)				.002
Less than HS^c^	40 (18)	29 (19)	11 (15)	
HS diploma or GED^d^	67 (30)	44 (30)	23 (31)	
Some college or more	109 (49)	76 (51)	33 (45)	
Unknown	7 (3)	0 (0)	7 (9)	
Income level (US $), n (%)				.02
<20,000	95 (43)	65 (44)	30 (41)	
20,000-39,999	35 (16)	22 (15)	13 (18)	
40,000-59,999	19 (9)	14 (9)	5 (7)	
>60,000	28 (13)	21 (18)	7 (9)	
Unknown	11 (5)	0 (0)	11 (15)	
Prefer not to answer	35 (16)	27 (18)	8 (11)	
Employment, n (%)				<.001
Employed full-time	73 (34)	52 (35)	21 (28)	
Employed part-time	26 (12)	14 (9)	12 (16)	
Disabled, cannot work	31 (14)	19 (13)	12 (16)	
Not employed	52 (23)	45 (30)	7 (9)	
Retired	23 (10)	13 (9)	10 (14)	
Unknown	18 (8)	6 (4)	12 (16)	
CR indication, n (%)				
AMI^e^	87 (39)	53 (36)	34 (46)	.13
CABG^f^	12 (5)	6 (4)	6 (8)	.20
Valve replacement or repair	18 (8)	13 (9)	5 (7)	.61
PTCA^g^ or stent	96 (43)	54 (36)	44 (59)	.001
Stable HF^h^	56 (25)	41 (28)	15 (20)	.24
Stable angina	5 (2)	5 (3)	0 (0)	.11
PHQ-9^i^ score >4	87 (52)^j^	74 (51)	13 (18)	.34

a *T* tests are used to compare means across 2 groups. Chi-square tests are used to compare proportions across 2×2 groups, with Fisher tests for comparisons across tables larger than 2 groups.

b CR: cardiac rehabilitation.

c HS: high school.

d GED: general equivalency degree.

e AMI: acute myocardial infarction.

f CABG: coronary artery bypass graft.

g PTCA: percutaneous transluminal coronary angioplasty.

h HF: heart failure.

i PHQ-9: Patient Health Questionnaire 9.

j Total PHQ-9 tests gathered at baseline (n=167).

**Figure 2.**
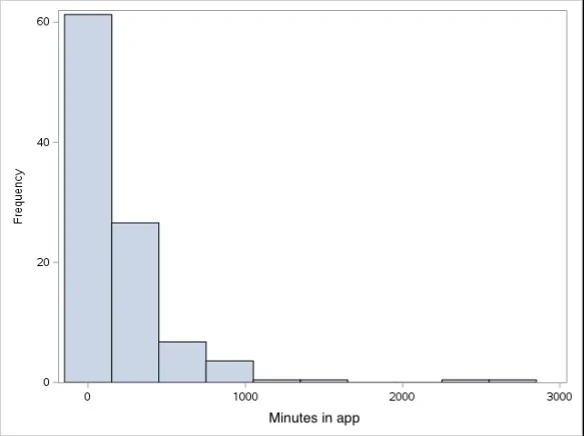
Histogram of app usage in minutes.

**Figure 3.**
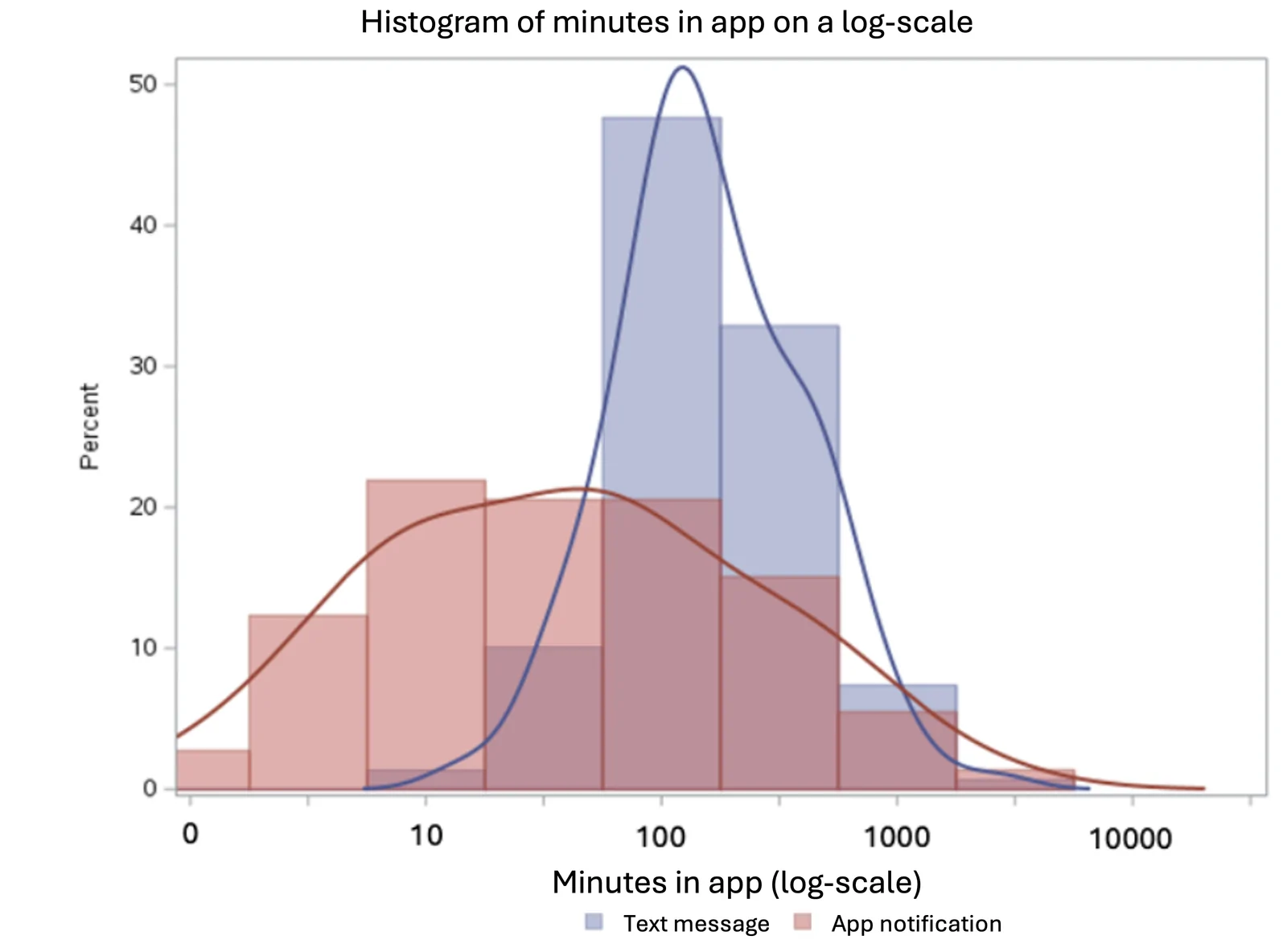
Histogram of log-transformed minutes spent in the app. Red represents patients who received app notifications only, and blue represents patients who received both text messages and app notifications. Overall, patients who received text message nudges used the app more than those who received app notifications only.

### Overall Predictors of Engagement

Unemployed participants spent 308 (95% CI 224-423) minutes in the app compared to 198 (95% CI 157-250) minutes spent by employed participants (unadjusted ratio 1.6, 95% CI 1.1-2.3; *P*=.03) (Table 2). Participants who had a qualifying diagnosis of myocardial infarction, non-ST–elevation myocardial infarction, or unstable angina spent 275 (95% CI 215-352) minutes in the app compared to those without (182, 95% CI 149-222 min; *P*=.01). Patients with a qualifying diagnosis of stable HF spent 142 (95% CI 104-193) minutes in the app compared to 244 (95% CI 204-291) minutes among those with other qualifying diagnoses (including but not limited to percutaneous coronary intervention or acute coronary syndrome; Figure 2 and Table 2).

**Table 2.** Minutes spent in the app by demographic subgroups.

Characteristic	Overall (min), mean (95% CI)^a^	App notification (min), mean (SD)^b^	Text message (min), mean (SD)^b^	Unadjusted ratio (95% CI)^c^	Unadjusted *P* value^c^	Adjusted ratio (95% CI)^d^	Adjusted *P* value^d^
Age tertile (y)							
20‐51	202 (153-265)	197 (594)	203 (377)	Reference	Reference	Reference	Reference
52‐61	215 (165-279)	130 (218)	272 (238)	1.1 (0.7-1.6)	.75	1.2 (0.8-1.7)	.39
62‐85	239 (181-314)	188 (303)	264 (275)	1.2 (0.8-1.7)	.40	1.3 (0.9-1.9)	.22
Sex							
Male	205 (171-246)	178 (399)	220 (227)	Reference	Reference	Reference	Reference
Female	258 (189-351)	127 (194)	313 (454)	1.3 (0.9-1.8)	.21	1.0 (0.7-1.5)	.92
Race							
White	236 (195-284)	273 (362)	178 (398)	Reference	Reference	—^e^	—
Black	174 (118-256)	72 (125)	203 (188)	0.7 (0.5-1.1)	.17	—	—
Other	181 (75-434)	46 (8)	235 (114)	0.8 (0.3-1.9)	.56	—	—
Unknown	189 (121-295)	185 (159)	213 (162)	0.8 (0.5-1.3)	.37	—	—
Ethnicity^a^							
Hispanic or Latino	227 (177-291)	165 (231)	257 (392)	1.1 (0.8-1.5)	.74	—	—
Non-Hispanic or Latino	215 (175-263)	167 (435)	238 (235)	Reference	Reference	—	—
Primary language							
English	204 (171-245)	162 (395)	225 (233)	Reference	Reference	Reference	Reference
Spanish	261 (191-357)	174 (257)	307 (465)	1.3 (0.9-1.8)	.18	1.5 (1.02-2.29)	.04
Site of CR^f^							
Hospital-based	209 (173-253)	181 (423)	224 (318)	Reference	Reference	Reference	Reference
Home-based	237 (181-310)	128 (150)	282 (281)	1.1 (0.8-1.6)	.46	1.1 (0.8-1.5)	.77
Level of education							
Less than high school	172 (120-248)	93 (135)	202 (209)	Reference	Reference	Reference	Reference
High school diploma or GED^g^	171 (129-226)	55 (70)	231 (219)	1.0 (0.6-1.6)	.97	1.2 (0.7-1.9)	.49
Some college or more	259 (208-323)	237 (508)	269 (373)	1.5 (1.0-2.3)	.06	2.1 (1.3-3.4)	.003
Unknown	301 (126-718)	301 (273)	—	1.7 (0.7-4.5)	.25	3.0 (1.1-7.8)	.03
Employment							
Employed	198 (157-250)	162 (292.5)	216 (234.5)	Reference	Reference	—	—
Disabled, cannot work	148 (98-224)	62 (76.3)	202 (217.3)	0.7 (0.5-1.2)	.23	—	—
Not employed	308 (224-423)	396 (942.1)	294 (430.4)	1.6 (1.0-2.3)	.03	—	—
Retired	193 (119-311)	118 (182.1)	250 (235.4)	1.0 (0.6-1.7)	.91	—	—
Unknown or declined	224 (131-386)	182 (249.7)	310 (275.2)	1.1 (0.6-2.0)	.68	—	—
Income level (US $)							
<20,000	190 (150-241)	95 (135)	234 (238)	Reference	Reference	—	—
20,000-39,999	247 (167-364)	323 (706)	201 (128)	1.3 (0.8-2.0)	.26	—	—
40,000-59,999	122 (72-208)	7 (7.8)	164 (157)	0.6 (0.4-1.1)	.14	—	—
>60,000	278 (180-430)	282 (471)	277 (353)	1.5 (0.9-2.4)	.13	—	—
Declined to answer	258 (184-363)	167 (247)	323 (509)	1.4 (0.9-2.1)	.14	—	—
PHQ-9^h^ score							
≤4	277 (225-341)	264 (291)	279 (388)	Reference	Reference	—	—
>4	236 (193-288)	397 (667)	208 (196)	0.9 (0.6-1.1)	.27	—	—
Total	219^i^ (—)	167 (365)	245 (306)	1.5 (1.1-2.1)	.02	1.8 (1.3-2.6)	.001

a Reflects least-squared mean from a negative binomial regression across levels of the covariate, with a 95% CI for the mean.

b Reflects the arithmetic mean and SD of minutes among participants in that subgroup.

c Unadjusted ratio and *P* value reflect the unadjusted analysis of time in the app across levels of that covariate.

d Adjusted ratio and *P *value reflect results from the adjusted negative binomial model with a log-link function, accounting for age tertile, sex, preferred language, site of CR, level of education, and app notification vs text message nudges.

e Not applicable.

f CR: cardiac rehabilitation.

g GED: general equivalency degree.

h PHQ-9: Patient-Health Questionnaire-9.

i SD 328.

### Unadjusted App Notification vs Text Message

Patients receiving nudges as text messages spent 245 (SD 306) minutes in the app compared to patients receiving app notifications, who spent 167 (SD 365) minutes in the app (unadjusted ratio of minutes in the app among patients receiving both text messages and app notifications vs app notifications only: 1.5, 95% CI 1.1-2.1; *P*=.02).

### Adjusted Analyses

In an analysis adjusted for age category, sex, primary language, CR location, and highest education achieved, patients receiving text messages and app notification nudges spent an adjusted ratio of 1.8 (95% CI 1.3-2.6; *P*=.001) times as many minutes in the app compared to those receiving only app notifications (Table 2). The adjusted model also suggested that there was greater app use among patients who preferred Spanish (261 vs 204 min among those who preferred English; adjusted ratio 1.5, 95% CI 1.02-2.26; *P*=.04) and had at least some college-level education compared to participants with less than a high school education (262 vs 172 min; adjusted ratio 2.1, 95% CI 1.3-3.3; *P*=.002).

## Discussion

### Principal Findings

This analysis of a pragmatic study of app-based remote CR at an urban safety-net hospital aimed to identify correlates of app use and characterize app use among patients receiving nudges as text messages and app notifications or app notification nudges only. Our analysis of 223 patients demonstrated that app use was heterogeneous and heavily skewed toward low use. In an adjusted model, we found that increased use was associated with higher education and Spanish language–preferring patients. Most importantly, nudges sent as text messages and app notifications were associated with greater app use compared to nudges sent as app notifications in both adjusted and unadjusted models.

The results of this study suggest that patients who prefer Spanish and those with higher education levels spent more time in the app. In contrast to prior work, we did not find that older patients engaged less with the intervention [13-15]. Other studies have previously identified ethnicity and education level as important influences on patient engagement in chronic disease management [14]. It is important to note that most trials underrepresent the target population of this study, as most trials enroll participants from major university centers [16-18]. While this single-center study is affiliated with a major university, the study was entirely conducted at an urban safety-net hospital, suggesting that with adequate technology training, these adversely resourced patients may be interested in and engaged with the resources available to them. In the adjusted multivariate model, patients with higher levels of education spent more time in the app. Several prior studies have identified the level of education as an important mediator of socioeconomic differences in cardiovascular disease and CR participation, and our study replicated this finding among patients participating in virtual or hybrid CR [19,20]. In addition, the finding that Spanish-preferring patients spent more time in the app could suggest 2 disparate possibilities: that patients need more time to navigate the interface, or they are spending more time in the app to learn more about their condition. There is little prior work in this area, and this warrants further investigation.

While most studies in this realm report changes in adherence, physical activity, or quality of life, few report the use of the mHealth platform itself [8,21]. Only occasional reports exist regarding the duration of wearables worn [22], time spent in mobile apps, or web logins [23,24] for the variety of remote CR platforms tested thus far. These smaller studies demonstrate high usability and acceptability, with use metrics ranging from 20 to 120 patients and 30 days to 24 weeks [9,25]. Other digital health interventions aimed at improving patient health found baseline technology use and intent for lifestyle change to be associated with patient engagement. Multiple studies have also found dose-response effects, with higher use among patients associated with greater improvement in clinical parameters [26-30]. A multicomponent health promotion intervention to promote weight loss identified that phone calls were deemed helpful by participants, but website and app usage were low [31]. Some studies suggest self-segregating cohorts of users, including high users, high users who decline, and low users [29,30]. This suggests significant variation over time, a phenomenon that should be studied further to identify strategies to keep patients engaged. Needs assessments and surveys with engaged patients have identified personalization as a suggested strategy to maintain engagement [32]. Some qualitative studies report nonadopters among an aging population, with “forgotten passwords” and “outdated technology” reported as obstacles to use, but these studies represent a limited subset of perspectives [33,34]. A mixed methods study demonstrated low adherence across all demographic subgroups and identified fear of movement and exercise self-efficacy as major obstacles to a home CR intervention among patients with HF [13]. Significant heterogeneity is demonstrated in these studies, suggesting that much work needs to be done to understand how to best augment adherence to home-based or virtual CR alternatives.

It is crucial to study strategies that may augment use or adherence among patients to virtual alternatives, given that greater use may result in better clinical outcomes. One CR study compared changes in activity among patients receiving text message reminders for activity, finding variation in steps walked based on the device used for measurement and the number of days since study enrollment [35]. However, the majority of studies investigating strategies to augment patient engagement in mHealth are outside of CR. In a review of interventions to augment engagement with mHealth, strategies included incorporating behavior change theory, reminders, goal-setting, automatic enrollment, and provider-led strategies [26,36]. A greater number of behavior change theories incorporated may have led to greater behavior change, but few studies referenced theories, and the effects were no different from the overall studies in the review. Provider-facilitated engagement led to the greatest engagement, but no reasons were provided for this or for the high attrition with other engagement strategies. Another study identified that opt-out consent led to greater participation in a mHealth intervention to improve medication adherence among groups that have been historically underrepresented [16]. We found a higher association of app use among patients receiving text message nudges, suggesting that this intervention factor may modify use among patients. While our analysis cannot determine causality, this is hypothesis-generating regarding strategies to better engage patients in mHealth interventions. Given the lack of standard measurements, and variations in follow-up periods, it remains difficult to compare use among studies or strategies. Without examining or comparing use, it will remain unclear which patients require additional support. Mobile interventions require careful implementation and theoretical bases to ensure that we are able to reach all populations, given ongoing concerns regarding widening disparities with the increasing advent of digital health interventions [16,17,21,37-39]. To deliver CR as a mobile or remote option, quality cannot be compromised, and patients must be supported and engaged [1]. Ongoing work is necessary to determine how to best engage patients.

### Limitations

Limitations of this study include that it was a single-center study that excluded patients who did not speak English or Spanish. The nonrandomized, pre-post design used in the analysis additionally precludes the determination of causation. Given the temporal differences in study arms, there are several factors that this study design could not account for, such as improved training protocols, study staff experience, or seasonality. We conclude that nudge delivery modality influences patient engagement, with text messaging being associated with greater engagement than app notifications. Furthermore, this is an intermediary assessment. The true value of increased time in the app will be determined through an analysis of patient outcomes. Strengths of this study include the 223 patients, which is a larger sample size than many studies exploring similar questions.

### Conclusions

We conclude that an app-based CR platform at an urban safety-net hospital was feasible and acceptable. Spanish language–preferring patients and those with higher educational status demonstrated greater use of the app. Most importantly, nudges delivered as text messages and app notifications were associated with greater use of the app platform compared to patients who received nudges as app notifications alone. It remains to be seen how best to support and engage patients who demonstrated lower app use, and whether this will impact outcomes within the study. As we transition to incorporating hybrid or virtual CR models into care, it is crucial that we identify strategies to better engage patients who have been historically excluded. These findings inform future interventions, which may consider text messages to engage patients more effectively than app notifications. Further research remains necessary to evaluate how to support patients who engage less with the app, such as those with lower educational status, and to assess whether app use correlates with patient outcomes such as CR adherence or functional capacity.

## Supplementary material

10.2196/64502 Checklist 1 STROBE checklist.

## Abbreviations

CR: cardiac rehabilitation
ERA-Nudge: Enhancing Rehabilitation Adherence Through Optimized Nudge
HF: heart failure
mHealth: mobile health
PHQ-9: Patient Health Questionnaire-9
STROBE: Strengthening the Reporting of Observational Studies in Epidemiology
